# *Pseudomonas aeruginosa* Implant-Associated Bone and Joint Infections: Experience in a Regional Reference Center in France

**DOI:** 10.3389/fmed.2020.513242

**Published:** 2020-10-26

**Authors:** Matteo Cerioli, Cécile Batailler, Anne Conrad, Sandrine Roux, Thomas Perpoint, Agathe Becker, Claire Triffault-Fillit, Sebastien Lustig, Michel-Henri Fessy, Frederic Laurent, Florent Valour, Christian Chidiac, Tristan Ferry

**Affiliations:** ^1^Hospices Civils de Lyon, Lyon, France; ^2^Université Claude Bernard Lyon 1, Lyon, France; ^3^Centre Interrégional de Référence des Infections Ostéo-articulaires complexes (CRIOAc Lyon), Hospices Civils de Lyon, Lyon, France; ^4^Centre International de Recherche en Infectiologie, CIRI, Inserm U1111, CNRS UMR5308, ENS de Lyon, UCBL1, Lyon, France

**Keywords:** pseudomonas, osteomyelitis, ciprofloxacin, implant-associated bone infections (IABI), bone and joint infection

## Abstract

**Background:** *P. aeruginosa* implant-associated bone and joint infections (BJI) is considered to be one of the most difficult to treat BJI. The data focusing specifically on this pathogen are sparse, and it seems difficult to extrapolate the results obtained with *Enterobacteriaceae*.

**Methods:** We performed a retrospective observation study of all *P. aeruginosa* implant-associated BJI diagnosed at our institution from 2011 to 2018. We defined failure as any type of relapse, including persistence of the same *P. aeruginosa*, superinfection by another organism(s) or any other cause of relapse such as the need for a subsequent surgery. Nonparametric statistical methods were used to compare the study groups and Kaplan-Meier curves and multivariate Cox analysis and were used to detect determinants associated with treatment failure.

**Results:** A total of 90 patients (62% men, median age 60 years IQR 47–72) including 30 (33%) prosthetic-joint infections and 60 (66%) other implant-associated BJIs were studied. Most of them were acute (62%). During the prolonged follow-up, (median 20 months; IQR 9–37), 23 patients (26%) experienced treatment failure. Optimal surgical treatment (DAIR for acute forms, explantation, 1-stage or 2-stage exchange for others) was significantly associated with a higher success rate in the univariate analysis (*p* = 0.003). Sixty-four (71%) patients received effective initial treatment against *P. aeruginosa* administered and 81 of them (90%) did for at least 3 weeks: both these parameters correlated with a higher success rate. In the multivariate Cox-analysis optimal surgical treatment, IV effective treatment of at least 3 weeks and treatment with ciprofloxacin for at least 3 months proved to be independently associated to a better outcome in patients with *P. aeruginosa* implant-associated BJI.

**Conclusion:** *P. aeruginosa* implant-associated BJI is one of the most difficult-to-treat BJI, with a strong impact on the prognosis of the surgical strategy. An effective initial IV antibiotic treatment for at least 3 weeks seems to be required, followed by oral ciprofloxacin for a total duration of 3 months.

## Introduction

Implant-associated bone and joint infection (BJI) is an uncommon, but dreadful complication of arthroplasties and orthopedic trauma. Despite technological and medical effort in preventing such conditions, the amount of implant-associated infections is growing because of the increasing number of implant devices (1, 2). According to Zimmerli et al., the infection rate during the first 2 years varies according to the site and it is <1% in hip and shoulder prostheses, <2% in knee prostheses, and <9% in elbow prostheses (3). With regard to internal fixation devices, about 5–10% becomes infected, with a significant disproportion between the major rate of infection after internal fixation of grade 3 open fractures (which may exceed 30%) against the 0.5–2% rate of infection after internal fixation of closed wounds (1).

The most frequently isolated microorganisms in implant-associated BJI are Gram-positive cocci, with *Staphylococcus aureus* being the most recurrent cause, while Gram-negative bacteria (GNB) are responsible for 10–23% of all episodes, causing most often acute and polymicrobial infections (1, 3–6). Even if GNB cause a minor- yet, substantial- proportion of all implant-associated BJI, they draw the attention of the medical community in light of the fact that the treatment is rather complicated and they show a less optimal outcome with longer hospitalizations -and higher costs- due to their peculiar virulence, their growing resistance to antibiotics and the comorbidities of the patients they usually infect, generally immunocompromised ones (6–9). *P. aeruginosa* is a particular GNB, commonly considered as non-fermenting bacterium, that causes 5 to 20% of the GNB infections, and recent data revealed that 14% of patients with open fracture suffered from *P. aeruginosa* infection (10, 11). *P. aeruginosa* is considered as one of the most difficult-to-treat GNB, as a result of its growing rate of multidrug-resistant strains and its ability to develop particular virulence and persistence mechanisms, such as biofilm formation and production of small colony variants (12).

Treatment strategies for staphylococcal implant-associated BJI are somewhat standardized, with a clear percentage of success, since they represent a significant cause of infection, which makes them easier to sample and study (1, 13, 14). On the contrary, our path to mastering Gram-negative implant-associated infections has been paved with scarce published experience, mostly retrospective studies, which showed inconsistent data concerning surgical and antimicrobial treatment (6–8, 15–18). Currently, guidelines for antibiotic treatment of GNB implant-associated infections recommend beta-lactams and ciprofloxacin (1, 13). This has also been supported by a large multicentre study which deals with acute GNB infections treated with debridement antibiotics and implant retention (DAIR), reporting a 79% success rate in ciprofloxacin-susceptible GNB PJI (19). In this very same setting, *P. aeruginosa* caused up to 20% of the GNB infections (19). Of note, none of these studies focused specifically on *P. aeruginosa* infections.

To our knowledge, along the years there have been just a few publications with *in vitro* studies supporting the role of fluoroquinolones against *P. aeruginosa* (20–22). However, some antimicrobial combinations, such as cefepime-ciprofloxacin and ceftazidime-ciprofloxacin, have been reported as successful options in the treatment of *P. aeruginosa* bone and joint infections (7, 16). Moreover, ciprofloxacin has been connected to a better treatment outcome when administered in case of susceptible GNB (15, 17, 23) and also of *P. aeruginosa* (19, 24).

The aims of the present study are to review our experience with the treatment of acute, delayed or chronic implant-associated *P. aeruginosa* BJI, and to analyze the impact of optimal surgical treatment, effective antimicrobial IV therapy and ciprofloxacin use on the prognosis.

## Materials and Methods

### Study Design and Population

We performed a retrospective study at the Croix Rousse hospital (Hospices Civils de Lyon, France), that is the national French reference center for osteoarticular infections of the South-East region (CRIOAc Lyon; http://www.crioac-lyon.fr). We included all patients, independently of time on follow-up, with *P. aeruginosa* implant-associated infection managed in our institution between January 2011 and June 2018. All cases present in this cohort were discussed and handled thanks to the cooperation of our multidisciplinary group. Data were obtained from the electronic and written medical records, collected into a Microsoft Access Database. This study is subject to declaration with the local Commission for Data Protection and Liberties under the n°18-176 and is registered on ClinicalTrial under the n°NCT03624855.

### Definitions

Implant-associated infection caused by *P. aeruginosa* was diagnosed according to the definition of organ/space surgical site infection proposed by the CDC (25) and also fulfilled the IDSA definition for patients with PJI and the new definition proposed by Metsemakers et al. for patients with internal fixation associated infections (13, 26). We identified as hematogenous acute BJIs those cases in which the patient had a normal joint function after the implantation, but experienced a sudden onset of symptoms more than 3 months after the index surgery, as previously reported by Wouthuyzen-Bakker et al. (27). At least one positive sample with *P. aeruginosa* in culture from deep perioperative samples was required.

Implant-associated infections in this study were defined as “early” if they occurred within 1 month from the date of implantation, “delayed” if they occurred between 1 and 3 months from the date of implantation and “chronic” if the onset of symptoms was >3 months from the date of implantation.

Treatment failure was defined as any type of relapse of implant-associated infection including persistence (new surgery with *P. aeruginosa* in culture), superinfection [isolation of another organism(s)] or any other cause of relapse such as the need for a subsequent surgery. Treatment was considered successful if the infection was in remission at the end of the course of antibiotics and during the entire usual follow-up in our institution. In case of need, suppressive therapy was undertaken for the treating physician to prolong the antibiotic treatment indefinitely in patients at high risk of persistence and relapse.

Optimal surgical treatment was evaluated according to the type of surgery and the timing of the infection. In case of acute infections, we defined “DAIR” as an optimal choice of intervention if performed within 1 month following the date of implantation and for patients with hematogenous infection. If the *P. aeruginosa* implant-associated BJI was itself a superinfection on an implant previously infected by another microorganism, and if the current episode of the infection was asymptomatic and discovered accidentally on systematic bone biopsies (i.e., without clinical signs of infection), we reckoned the surgical treatment as optimal independently from the timing. While if the superinfection was accompanied by the onset of new clinical symptoms or by the worsening of the patient conditions, we assessed as optimal only the surgical treatment which was undertaken within 1 month from the previous surgery.

Effective initial antibiotic treatment against *P. aeruginosa* was defined by the use of an active IV beta-lactam drug, based on drug-susceptibility on the antibiogram.

According to the classification of the Common Terminology Criteria for Adverse Events (CTCAE), serious adverse events (SAE) were defined as CTCAE grade 3–5 (28). All SAE were reviewed by a pharmacist and were attributed (or not) to the antibiotic on *P. aeruginosa*.

### Statistical Analysis

Descriptive statistics were used to estimate the frequencies of the study variables, described as effective (%) for dichotomous values and medians [interquartile range (IQR)] for continuous values. For the percentage calculation of each variable, the number of missing values was excluded from the denominator. Nonparametric statistical methods were used to compare the study groups (chi-square test, Fisher's exact test, or Mann-Whitney U test, as appropriate). Univariate Cox analysis and Kaplan-Meier curves (using the log-rank test) were used to determine determinants associated with treatment failure. Multivariate Cox analysis that includes significant determinants identified in the univariate analysis was performed, by adopting a ratio of 10 events per independent variable to avoid overfitting (maximum of three variables in the present study, selection based on the univariate analysis). A *p*-value of <0.05 was considered significant. Statistical analyses were performed using SPPS Statistics Base 17.0 (Softonic International, San Francisco, CA, USA).

## Results

Among the 1,638 implant-associated BJI occurring over the 7-year study period, 90 patients (5.5%) from the beginning of 2011 to end of 2017 were infected by *Pseudomonas aeruginosa* (including 30 with a PJI) according to our definition and were included. Basic demographic information can be found in Table 1.

**Table 1:** Characteristics of the 90 patients with *P. aeruginosa* implant-associated BJI according to the outcome.

**Characteristics**	**Whole population (*n* = 90)**	**Failure (*n* = 23)**	**Remission (*n* = 67)**	***p*^**a**^**
Age in years (median, IQR)	60 (47–72)	61 (43–74)	59 (47–72)	0.90
Male sex (*n*, %)	56 (62)	17 (74)	39 (58)	0.18
BMI ≥ 30 (*n*, %)	24 (28)	6 (29)	18 (29)	1
Active smoking (*n*, %)	29 (35)	10 (44)	19 (32)	0.34
Score ASA > 2 (*n*, %)	30 (34)	8 (35)	22 (33)	0.90
Score Charlson > 4 (*n*, %)	24 (27)	7 (30)	17 (25)	0.64
Previous infection at the same site (*n*, %)	19 (21)	6 (26)	13 (19)	0.50
Prosthesis (*n*, %)	30 (33)	7 (30)	23 (34)	0.73
Age of implant in days (median, IQR)	47 (21.7–247.5)	40 (21–222)	63 (26–798)	0.29
**Type of infection (*****n*****, %)**				
Acute	56 (62)	14 (61)	42 (63)	0.98
Sub-acute	8 (9)	2 (9)	6 (9)	
Chronic	26 (29)	7 (30)	19 (28)	
Polymicrobial infection (*n*, %)	66 (73)	18 (78)	48 (71)	0.54
BJI due to *P. aeruginosa* ciprofloxacin-resistant (*n*, %)	11 (12)	9 (39)	2 (3)	<0.001
Optimal surgical treatment^b^ (*n*, %)	54 (64)	9 (39)	45 (72)	0.004
Effective initial IV treatment^c^ (*n*, %)	64 (71)	12 (52)	52 (77)	0.020
Treatment with ciprofloxacin^d^ (*n*, %)	79 (88)	13 (57)	66 (99)	<0.001

*IQR, interquartile range*.

a *The p-value was determined by using chi-square or Fisher's exact test for categorical variables, Mann-Whitney U test for continuous variables*.

b *After exclusion of the five patients who finally received suppressive antimicrobial therapy*.

c *Such as piperacilline, piperacilline-tazobactam, ceftazidime, cefepime, imipenem-cilastatin, ceftolozane-tazobactam, ceftazidime-avibactam, based on the susceptibility on the antibiogram*.

d *After exclusion of the two patients that received ciprofloxacin as suppressive therapy*.

Twenty-five patients experienced 28 adverse events during a course of treatment with antibiotics that were active on *Pseudomonas aeruginosa* (13 were SAE, 16 caused the interruption of the effective treatment, while six of them occurred in the ending phase of treatment and shortened merely the course of antibiotic hopefully without nicking the quality of the medical therapy).

Sites of infection were: knee (16), spine (15), hip (15), tibia (8), jaw (7), skull (6), ankle (5), femur (4), elbow (2), shoulder (2), foot (2), calcaneum (2), others (2), pubis (1), sacroiliac bone (1), humerus (1), patella (1), heel (1).

**Figure 1:**
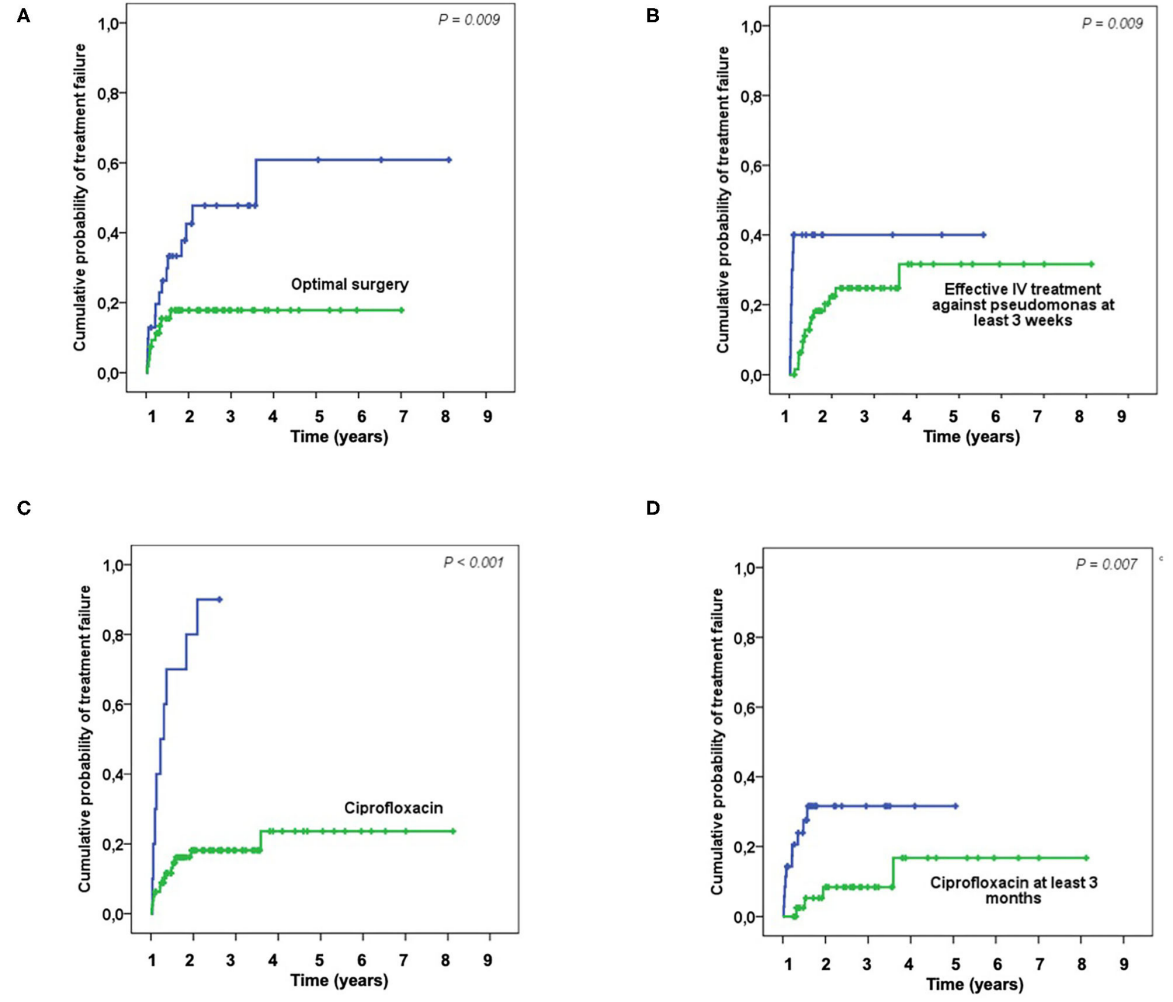
Kaplan-Meier curves showing the probability of treatment failure depending on the surgical and medical treatments: Optimal surgery **(A)**; Effective IV treatment against pseudomonas at least 3 weeks **(B)**; Treatment with ciprofloxacin **(C)**; Treatment with ciprofloxacin at least 3 months **(D)**.

Fifty-eight (64%) patients were considered to have optimal surgical treatment, including 21 DAIR for an acute infection, 2 had incomplete implant removal, 31 complete implant removal for a chronic infection, one complete ablation followed by amputation and one DAIR followed by amputation. Among the thirty-two (36%) patients who do not meet these criteria, 20 had DAIR for a delayed or chronic infection, 1 had an incomplete implant removal, 13 had a complete implant removal. During a prolonged follow-up [median follow-up of 20 months (IQR, 9–37)]; 24 patients without failure were followed at least 2 years, 23 patients experienced a treatment failure: seven patients experience a persistence of *P. aeruginosa* after treatment, while 16 had a superinfection caused by another organism(s). Of note, 40 patients were lost to follow-up during the first 2-years, but these patients were not excluded in the final analysis. Optimal surgical treatment was significantly associated with a higher success rate in the univariate analysis (*p* = 0.003) and in the Kaplan-Meyer survival curve (log-rank test, p=0.009) (Table 1; Figure 1A). As long as it concerns the antimicrobial treatment, sixty-four (71%) patients received effective initial treatment against *P. aeruginosa* administered by IV, while 26 (29%) did not. Two patients with MDR *P. aeruginosa* (17%) received ceftolozane/tazobactam or ceftazidime/avibactam. Not receiving an effective initial IV drug exposed the patient to an early failure (blue line in Figure 1B) and when we considered an IV treatment of at least 3 weeks, which was undertaken by 90 (81%) patients, we found that it correlates with a higher success rate both in the univariate analysis (*p* = 0.020) and according to the Kaplan-Meyer curve (log-rank test, *p* = 0.009) (Table 1, Figure 1B). Eleven (12%) patients had an infection due to a *P. aeruginosa* resistant to ciprofloxacin and this impacted as well (*p* < 0.001). In the end, we evaluated the effectiveness of the treatment with ciprofloxacin. Seventy-nine (88%) patients received a course of therapy with ciprofloxacin and we found this as significantly associated with a higher success rate in the univariate analysis (*p* < 0.001) (Table 1; Figure 1C). Moreover, we observed a higher risk of failure if patients received <3 months of ciprofloxacin (log-rank test, *p* = 0.007) (Figure 1D). In the multivariate Cox analyses, we included in the final model three variables which finally depict the optimal pattern of treatment: optimal surgical treatment, IV effective treatment of at least 3 weeks and treatment with ciprofloxacin for at least 3 months (Table 2).

**Table 2:** Multivariate Cox analysis that includes significant determinants for failure identified in the univariate analysis.

	**HR**	**95% CI**	***p***
Optimal surgical treatment^*^	0.32	0.11–0.98	0.045
IV effective treatment of at least 3 weeks^*^	0.15	0.004–0.054	0.003
Ciprofloxacin for at least 3 months^*^	0.23	0.07–0.75	0.015

*HR, Hazard ratio; 95% CI, 95% confidence interval*.

* *After exclusion of the five patients who finally received suppressive antimicrobial therapy*.

## Discussion

We have presented a case series of 90 implant-associated BJI caused by *P. aeruginosa* at our structure, managed at our institution during 2011–2017, which accounted for the 3% of all BJI in this 7-year experience. To our knowledge, this is the largest and only study about implant-associated infections due to *P. aeruginosa* along with the one of Shah et al., which was only focused on PJIs (29).

Our data show that these infections are mostly acute and often polymicrobial, possibly due to the high comorbidity index of the patients involved and the opportunistic nature of a *P. aeruginosa* infection (5, 6, 29). After a long-term follow-up, the remission rate of patients with a *P. aeruginosa* implant-associated BJI was 74% (67 out of 90), which is consistent with the results of Rodriguez-Pardo et al. on a smaller sample of *P. aeruginosa* cases (*n* = 43) included in a larger Gram-negative PJI study (19).

Surgical treatment is the cornerstone of Implant-associated infections and all of our patients underwent surgical procedures. Choosing the correct operation for the case among the number of options (DAIR, 1-stage or 2-stage exchange, palliative treatment) is much more subtle than what it looks like, and the decision should follow as possible the current guidelines. It must be a multidisciplinary, meticulous process, and it must take into account the patient status and integrate its functional prognosis in case of implant removal (13, 30). Lora-Tamayo et al. reported 33 patients with *P. aeruginosa* infected PJI and reached an overall success rate of 81% by treating early post-surgical and hematogenous infections with stable devices and good soft tissue conditions with DAIR, while they opted for an implant removal for the chronic cases (31). Ascione et al. described 11 cases of *P. aeruginosa* PJI, as part of a broader study, treated with DAIR (80% overall success rate) or 2-stage exchange for late infections (85% overall success rate) (32). Once more, Veltman et al. presented a study on 12 early post-operative *P. aeruginosa* PJI treated with DAIR, reporting a success rate of 66% (24). These data are rather promising and in accordance with guidelines instructions, yet in contrast with the biggest *P. aeruginosa* PJI study (102 episodes in 91 patients), which pointed out a 5-year cumulative incidence of failure of 50% when treating PS PJI and an especially worse outcome for those treated with DAIR (2 year cumulative survival free rate of 26%) (29). However, this study took into account infections occurred over a long period, therefore their optimal management was clearly limited by the lack of an established protocol, as proved by the fact that most patients who underwent DAIR had chronic infections (29). Among our patients the average duration of IV treatment was of 79 days [median 63 days, IQR (44–96)], while the average duration of the oral treatment with ciprofloxacin was of 111 days (median 79 days: IQR, 29–99). A recent study on 242 GNB PJIs, among which the 20% was caused by *P. aeruginosa*, DAIR was successful in 68% of cases, with an increase to 79% in ciprofloxacin-susceptible GNB PJI treated with ciprofloxacin (19). By judging the adequateness of all our patients' surgical treatment according to the current guidelines (13, 30), we found that optimal surgical treatment was significantly associated with a higher success rate, as previously reported in a study with *S. aureus* PJI (33).

Effective initial antibiotic betalactam treatment against *P. aeruginosa* proved to be a factor correlated with a better outcome (*p* = 0.020) in accordance to the guidelines and previous experiences (7, 13, 16, 34). Even if such antibiotics are recommended as initial therapy, their optimal duration is unclear. In patients with fluoroquinolone-susceptible *Enterobacteriaceae*, it is largely admitted that the duration of IV treatment could be shortened to 2 weeks (20, 23, 35). In the study of Rodriguez-Pardo et al., *P. aeruginosa* cases were treated for a median of 60 days, with a combination of antibiotics in half of them, mainly an antipseudomonal beta-lactam plus ciprofloxacin. The median duration of the intravenous therapy (i.e., of the beta-lactam) was 18 days (19). As *P. aeruginosa* is considered to be a more difficult-to-treat bacterium in comparison with *Enterobacteriaceae*, as it has been speculated by some authors (8, 18), it is difficult to translate the results obtained with these latter bacteria exclusively, or with a minority of *P. aeruginosa*.

The treatment with ciprofloxacin was a factor significantly associated with a better outcome in our study. In the study of Shah et al., that included patients with *Pseudomonas* PJI in a period of time during which ciprofloxacin was not widely used during initial therapy (only nine out of the 102 received ciprofloxacin), the rate of success was particularly low (26%) in patients treated with DAIR (29).

This finding is in line with what has already been suggested by Martinez-Pastor et al. (23), who examined GNB BJI and fluoroquinolones in general. As already proposed by Rodriguez-Pardo et al., this finding supports the idea that the success of treatment depends on the susceptibility to this antibiotic and its use rather than on the causative microorganism (19). In this study, 28 of the 43 *P. aeruginosa* cases received ciprofloxacin, for a median of 43 days. The overall success rate was 79% (33 of 42 cases), which increased to 88% (29 of 33) when only patients with ciprofloxacin were considered (19).

According to the literature, ciprofloxacin proved itself to be effective given its qualities (namely oral availability, diffusion into the bone, activity against biofilms) (20, 36). Concerning the optimal duration of the fluoroquinolone treatment in GNB BJI, it is probably ranged from 6 weeks−3 months, as we also found by checking the median duration of treatment in other studies (19, 24). In patients with fully susceptible *Enterobacteriaceae* native BJI, 6 weeks of treatment seem to be adequate. In patients with implant-associated BJI, a treatment course of 3 months has to be discussed, especially if *P. aeruginosa* is involved, as we found that such a duration was associated with a better outcome.

Of note, infection with a ciprofloxacin-resistant *P. aeruginosa* has a huge impact on the outcome: it has already been spotted as a risk factor advocating for implant removal even in acute infections (23), among the 11 patients infected with a ciprofloxacin-resistant *P. aeruginosa* in our study, nine experienced a failure. For this reason, fluoroquinolones should be avoided as empirical and initial therapy, yet they must be given only once having reduced the bacterial load, after a course of intravenous beta-lactam (15). There is no standard treatment for MDR GNB infection and *P. aeruginosa* is peculiarly challenging to treat, with scarce therapeutic options, that generally recur to combination of a new generation beta-lactam such as ceftolozane/tazobactam (37) or ceftazidime-avibactam (38) with colistin, which are inherently associated with high risk of toxic effects, while some *in vitro* and animal studies suggest a potential activity of the rifampin-colistin combination (39, 40).

Our work is an observational retrospective study that presents all the limitations implied by the inherent nature of this kind of study design. However, in the face of implant-associated infections, surgical and clinical management cannot be randomized; thus, observation studies are the best quality information we will ever have in this scenario. Secondly, it is crucial to focus accurately on patients with *P. aeruginosa* implant-associated BJI, as conclusions obtained with *Enterobacteriaceae* are not completely transposable. Finally, as *P. aeruginosa* implant-associated BJI is a potentially severe disease and as our center is a reference center for the management of BJI, we particularly try our best to follow these patient population. Even if the rate of lost to follow-up after 2 years was not negligible, very few data are lacking in our medical records, leading to interpretable results obtained from this study.

## Conclusions

*P. aeruginosa* implant-associated BJI is one of the most difficult-to-treat BJIs, with a strong impact on the prognosis of the surgical strategy. An effective initial IV antibiotic treatment for at least 3 weeks seems to be required, followed by oral ciprofloxacin for a total duration of 3 months.

## Data Availability Statement

The datasets generated for this study are available on request to the corresponding author.

## Ethics Statement

The studies involving human participants were reviewed and approved by Hospices Civils de Lyon Ethic Committee. Written informed consent from the participants' legal guardian/next of kin was not required to participate in this study in accordance with the national legislation and the institutional requirements.

## Author Contributions

MC wrote the first version of the manuscript. MC and TF performed the literature review. CB, AC, SR, TP, AB, CT-F, SL, M-HF, FL, FV, CC, and TF participated to the patient care. All authors contributed to the article and approved the submitted version.

## Conflict of Interest

The authors declare that the research was conducted in the absence of any commercial or financial relationships that could be construed as a potential conflict of interest.
